# Novel Test Fixture for Characterizing MEMS Switch Microcontact Reliability and Performance

**DOI:** 10.3390/s19030579

**Published:** 2019-01-30

**Authors:** Protap Mahanta, Farhana Anwar, Ronald A. Coutu

**Affiliations:** Department of EECE, Marquette University, Milwaukee, WI 53233, USA; protap.mahanta@marquette.edu (P.M.); farhana.anwar@marquette.edu (F.A.)

**Keywords:** Microswitch, Microcontact, Test Fixture, Micromachining, Reliability

## Abstract

In microelectromechanical systems (MEMS) switches, the microcontact is crucial in determining reliability and performance. In the past, actual MEMS devices and atomic force microscopes (AFM)/scanning probe microscopes (SPM)/nanoindentation-based test fixtures have been used to collect relevant microcontact data. In this work, we designed a unique microcontact support structure for improved post-mortem analysis. The effects of contact closure timing on various switching conditions (e.g., cold-switching and hot-switching) was investigated with respect to the test signal. Mechanical contact closing time was found to be approximately 1 μs for the contact force ranging from 10–900 μN. On the other hand, for the 1 V and 10 mA circuit condition, electrical contact closing time was about 0.2 ms. The test fixture will be used to characterize contact resistance and force performance and reliability associated with wide range of contact materials and geometries that will facilitate reliable, robust microswitch designs for future direct current (DC) and radio frequency (RF) applications.

## 1. Introduction

Microelectromechanical systems (MEMS) technology is widely used in applications ranging from sensing to switching technology due to its low cost, low power consumption, and small geometries [1]. Microswitches are an example of a MEMS technology that shows promising performances in direct current (DC) and radio frequency (RF) applications [2,3]. Excellent device attributes (i.e., low contact resistance ~1 Ω and near-zero power consumption ~0 W) and superior RF performance (i.e. low insertion loss ~0.2 dB and high isolation ~30 dB) play a key role for the microswitches to be considered as a better alternative than the conventional solid-state DC and RF switches [4,5]. However, reliability is of great concern for them to be ubiquitously used by the industry where the lifetime requirement is typically 1–10 billion cycles depending on the specific application [6].

**Figure 1:**
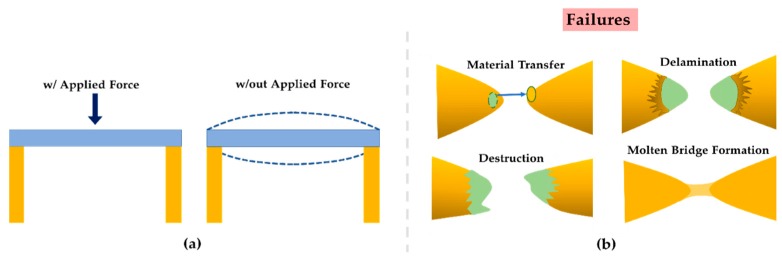
Microcontact failure mechanisms; (**a**) switching dynamics (contact force and contact bounce); (**b**) microcontact surface tribology (i.e., material transfer, delamination, degradation and bridge formation) after a certain number of switching cycles.

After billions of operations, microcontact area deterioration can severely impact the switching performance, which may ultimately cause device failure. Recently in [7,8], it has been demonstrated that material transfer, molten metal bridge (MMB), and contact delamination contribute to contact degradation after a certain number of switching cycles (Figure 1). Switching dynamics (e.g., contact force, contact closure time, and contact bounce), and microcontact surface tribology (e.g., contact resistance, contamination, adhesion, and material transfer) play the critical role in determining their reliability. A simple, quick, versatile, and efficient test fixture is required to study the contact surface tribology as well as to optimize the switching dynamics. Past studies have shown actual MEMS devices, modified nanoindentors, scanning probe microscopes (SPM), and atomic force microscopes (AFM) being used to perform microcontact reliability and performance studies. However, each of these test setups has limitations in microcontact data collection for showing acceptable device reliability. For example, an MEMS device-based test setup does not allow one to measure the contact force directly, and AFM, SPM, and nanoindentation are limited to cycle rates of 10–100 Hz [9,10,11,12]. 

In [13], Coutu et al. developed and assembled a simple and novel microswitch lifecycle test fixture. Nonetheless, difficulties in contact surface post-mortem analysis imposes the need to improve the microcontact support structure. Here, post-mortem analysis refers to microcontact surface failure (e.g., surface wear, contamination, and material transfer) investigation through Scanning Electron Microscopy (SEM), X-ray photoelectron Spectroscopy (XPS), Auger, micro Raman, etc. after a specific number of switching operations regardless of contact failure.

In this work, the design, analysis, and progress on a silicon on insulator (SOI) micromachined microcontact support structure are discussed. A fixed-fixed beam with an upper hemispherical contact bump was bonded on top of a set of gold (Au) pillars before any switching operations. After a desired number of cycling operations, the microcontacts were separated to evaluate the contact surface wear. Finite element analysis (FEA) has been performed to analyze the beam mechanics. A relation between the contact closure time and contact force has been developed for the fixed-fixed beam structure. The impact of closure time on various switching conditions (e.g., cold-switching and hot-switching) has been investigated theoretically with respect to the test signal. Furthermore, a condition for the minimum length of an Au pillar has been developed for the available applied force in the test system. The test fixture was used to characterize contact resistance, contact force, and adhesion force associated with wide range of contact materials. Engineered micro-electrical contacts were fabricated and tested using our novel test fixture for acquiring significant data to design a robust and reliable MEMS switch for future DC and RF applications. In addition, this test setup is capable of measuring micromachined membrane force versus deflection behavior, as well as sensing micro force exerted by biological cell movements. A force sensor integrated with a piezoelectric actuator can be used to apply known, calibrated forces in μN ranges onto the membrane for verifying its spring behavior [14,15].

## 2. Test Fixture Assembly with Improved Contact Support Structure

### 2.1. Test Fixture

**Figure 2:**
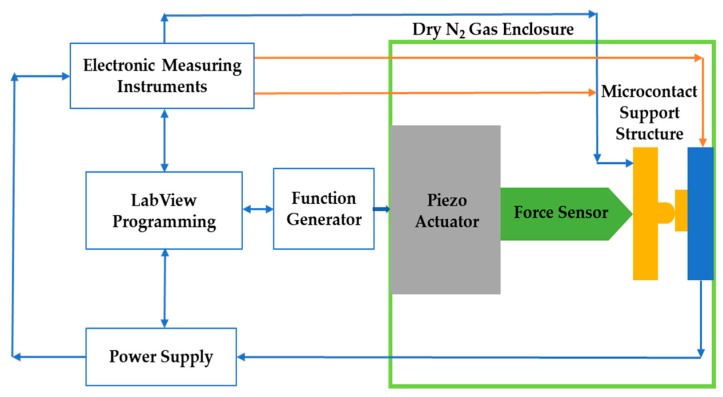
Schematic representation of the microcontact test fixture assembly.

The block diagram representation of the test fixture can be illustrated as Figure 2. 

The test fixture consists of two separate micromachined contacts, three piezoelectric actuators, a force sensor, an alignment stage, vibration isolation table, dry-box, and necessary electronic measuring instruments. In MEMS switches, mechanical movement of the device structure is used to achieve a short circuit or open circuit to provide switching functionality. In this test fixture, two separate micromachined contact support structures were incorporated, with one being a moveable contact (upper) and the other being a stationary (lower contact) to mimic actual MEMS switch operation. The piezoelectric actuator—along with a high sensitivity, high resonance force sensor—provided repeatable and precise position/force control in the contact region. The integration of a force sensor with the piezoelectric actuator facilitated the simultaneous measurement of contact force and contact resistance. In addition, since the applied force and the beam’s restoring force were known, adhesion force was the difference between these two forces during sensor withdrawal. We used Thorlab’s state-of-the-art Nanomax stage to provide nanometric positioning on three orthogonal axes. Each axis was controlled through modular piezoelectric actuators. In addition, a z-axis Joystick control provided extra intuitive, tactile, and manual positioning of the stage being driven. Initially the force sensor was placed at an arbitrary position (left/right) onto the beam using x-axis. Then, using y-axis and z-axis movement, we moved the probe tip forward/ backward and up/down. This special arrangement provided numerous options for reconfiguration and novel test parameters. The test fixture was housed in a custom “dry box” type enclosure to control the ambient environment and minimize surface contamination. For automation in testing and data collection, LabView programming software was utilized.

#### Experimental Setup for Piezoelectric Actuator and Force Sensor Calibration

**Figure 3:**
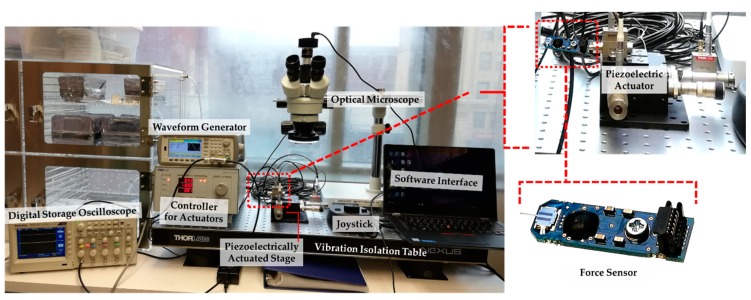
Experimental setup of the piezoelectric actuator and force sensor; inset shows the force sensor.

Figure 3 shows the experimental setup for calibrating individual and integrated performance of Thorlabs’ PAZ005 piezoelectric actuator (Thorlabs Inc., Newtown, NJ, USA) and FemtoTools’ FT-S1000 force sensor (FemtoTools, Buchs, Switzlerland) using a Thorlabs’ BPC-303 controller (Thorlabs Inc., Newtown, NJ, USA). Each of them was tested and calibrated prior to assembling into the test fixture. The piezoelectric actuator was integrated in the test fixture to provide 10–900 µN force with the displacement range of 0–20 µm. It has both open loop control and close loop control options, each with different speeds and accuracies. Close loop control is comparatively slower but more accurate than open loop control. Therefore, close loop control was appropriate for initial contact testing (ICT). Even though open loop control is faster, it suffers from hysteresis. While operating at maximum cycle rate, both control mode operations are greatly affected by the piezoelectric actuator’s capacitive load, circuit conditions, and system’s mass. This effect was minimized through a proportional-integral-derivative (PID) controller and a function generator. A force sensor with high sensitivity (i.e. μN resolution) and high resonance frequency was attached to the piezoelectric actuator to perform the test at cycle rates up to 5 kHz. This force sensor has a 50 μm × 50 μm square sensing area which was aligned to the other components in the fixture through the mounting/alignment fixture. 

### 2.2. Improved Microcontact Support Structure

#### 2.2.1. Contact Resistance Modeling

**Figure 4:**
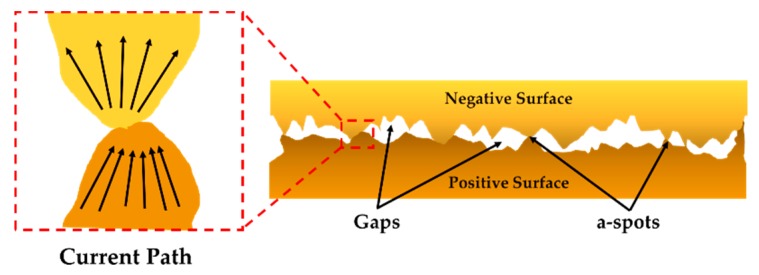
Contacting asperity peaks/ “a-spots” creating the conducting path for electrons.

In [16], the equation for constriction resistance was modelled without considering the contact surface contamination. This model is well suited for a smooth macro-contact surface. However, at the micro/nano scale, contact surfaces are not perfectly smooth, and, thus, surface contamination is significant at microcontact region [17,18]. At this scale, it has also been explored that the contact surfaces are formed from ridges and tops named asperity peaks or “a-spots,” which usually provide the conducting path for electrons (Figure 4). 

In [19], it was reported that area of the contact material is sensitive to both elastic and plastic deformation of such “a-spots”. They modelled the radius of an “a-spot” under elastic and plastic deformation as,

(1) $$r_{E}\;=\;\sqrt{\frac{2F_{c}R}{4È}}\;\;$$

(2) $$r_{P}\;=\;\sqrt{\frac{F_{c}}{H\pi }}\;$$

where $r_{E}$ is the contact radius due to elastic deformation, $r_{P}$ is the contact radius due to plastic deformation, $F_{c}$ is the contact force, *R* is the radius of curvature of the “a-spots”, *H* is the hardness of the material, and *È* is the Hertzian modulus of the contacting surfaces. 

One of the classical models for contact resistance has been developed through Maxwell’s spreading resistance theory and can be expressed as [16],

(3) $$R_{c}=\;\frac{\rho }{2r}\;$$

where ρ is the resistivity of the material and *r* is the radius of the “a-spots”. This model fits well with the constriction resistance model if the contaminant film resistance is not considered. Based on the diffusive electron transport theory, the resistance model for elastic and plastic deformation can be expressed as [10],

(4) $$R_{cDE}=\;\frac{\rho }{2}\;\sqrt[{3}]{\frac{4È}{3RF_{c}}\;}\;$$

(5) $$R_{cDP}=\;\frac{\rho }{2}\sqrt{\frac{H\pi }{F_{c}}}\;$$

where $R_{cDE}$ is the resistance for elastic deformation, $R_{cDP}$ is the resistance for plastic deformation, ρ is the resistivity of the material, *H* is the hardness of the material, $F_{c}$ is the contact force, *R* is the radius of curvature of “a-spots,” and *È* is the Hertzian modulus of the contacting surfaces. The resistance model associated with elastic deformation is only valid at low contact force region.

For our contact support structure, the resistance can be modelled as [20],

(6) $$R_{c}=\;R_{measurement}-R_{cf}-R_{sh}-R_{p}$$

where $R_{measurement}$ is the measured resistance value, $R_{cf}$ is the resistance due to contaminate film, $R_{sh}$ is the sheet resistance, and $R_{p}$ is the parasitic resistance generated from clip leads and solder connections. Generally, contaminant resistance is very low compared to the other resistance values. Therefore, we can approximate the model (6) as,

(7) $$R_{c}=R_{measurement}-R_{sh}-R_{p\;}$$

#### 2.2.2. Contact Voltage-Temperature Modeling

In microswitches, contact resistance increments with the increment of contact temperature results in contact failure. As the signal faces high power load during transitions, ‘hot’-switching phenomena has more impact on microcontact reliability than ‘cold’-switching [21]. In hot-switching, current density, temperature, and material transfer lead the microcontact into electrical failure. During contact opening, the a-spots with small contact areas constrict the current and lead to a significant joule heating at the contact interface. The contact voltage-temperature relationship at the contact region is expressed as [22],

(8) $$V_{c}{}^{2}=4L\;\left(T_{c}{}^{2}-T_{0}{}^{2}\right)\;$$

where $V_{c}$ is the voltage drop across the contact, *L* is the Lorenz constant (~$2.4\times 10^{-8}$ WΩ$K^{-2}$), $T_{c}\;$is the temperature in the contact, and $T_{0}$ is the bulk temperature. 

Now, rearranging the Equation (8) for the contact temperature, we have,

(9) $$T_{c}=\;\sqrt{\frac{V_{c}{}^{2}}{4L}+T_{0}{}^{2}}$$

Here, the contact voltage drop $V_{c}$ can be calculated using a known test current *i* as,

(10) $$V_{c}=iR_{c}$$

A rise in temperature around the contact area is considered as one of the prime issues in high-power applications as material softening occurs at this temperature. The softening, melting, and boiling voltage for Au is reported as 0.08 V, 0.43 V, and 0.88 V, respectively [20]. This temperature rise causes overheating, shrinking, and cracking of contact materials; thus, the contact may fail eventually. It has been reported that the average failure time for a microswitch with Au–Au contacts is relatively low because of its low hardness and low softening temperature [19]. Since the focus in this study is not on improving microcontacts, per se, but on a new microcontact support structure for testing contacts, gold is used for attesting the concept. For future contact material study, we will consider platinum (Pt), rhodium (Rh), ruthenium (Ru), carbon nanomaterials, and the metal alloys as contact materials, and Au will be used as benchmark material for comparing their performances.

#### 2.2.3. Beam Modeling, Testing Procedure and Contact Cycling

In the test fixture, the contact support structure is similar to the structure of a doubly fixed beam loaded at the center. The stiffness of the beam is calculated as,

(11) $$K=\;\frac{16\times E\times w\times t^{3}}{L^{3}}\;\;\;$$

where *E* is the Young’s modulus, *w* is the width, *t* is the thickness, and *L* is the length of the beam [23]. From (11), it is evident that shorter beams are stiffer than longer beams. For fabrication, it is easier to vary the length while keeping the other beam design parameters fixed. We investigated the microcontact behavior by varying the beam length at different contact force regions.

The beam resonance frequency sets the limit for highest mechanical cycling rate. The resonance frequency of a simply supported fixed-fixed Si beam structure can be expressed as,

(12) $$f=\;\frac{1}{2\pi }\sqrt{\frac{2K}{m}}$$

where *m* is the mass and *K* is the stiffness of the Si beam. 

The controlling of contact closure time (i.e. electrical and mechanical) relative to the test signal enables the study of contact surface wears under different switching conditions. Moreover, the speed of contact opening/closing may also enable the study of surface wears. For example, the molten metal bridge forms while the contact opening speed is slower, and surface delamination occurs while the contacts are opened abruptly [24]. 

**Figure 5:**
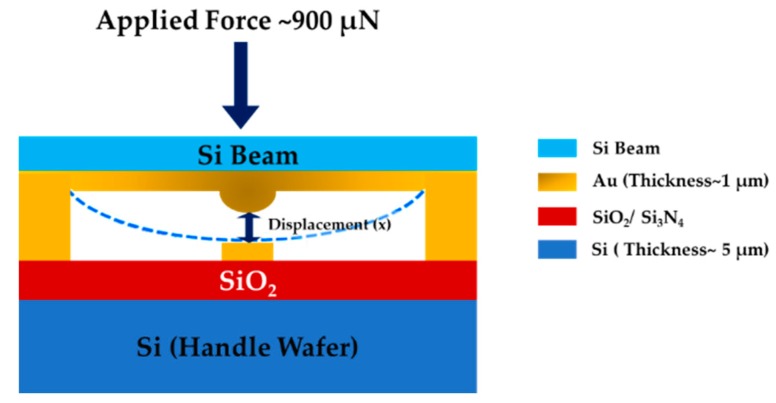
Schematic illustration of beam motion under available applied force.

To calculate the actuation rate and investigate the impact of its on contact surfaces, we have performed detailed timing analysis for the piezoelectric actuator as well as for the beam. The contact duration can be calculated by subtracting twice the time elapsed through the actuator and the mechanical Si beam from the actuation signal period. Actuator response time with respect to the actuation signal is expressed as,

(13) $$t_{r}=\;\frac{CV_{peak}}{I_{max}}$$

where *C* is the actuator’s capacitance, $V_{peak}$ is the maximum applied voltage, and $I_{max}$ is the maximum applied current for the actuator. As the piezoelectric material has a capacitance, the rise time can be regulated using range of applied voltages and currents. After elapsing this time with responding, the actuator starts to push the mechanical Si beam to bend until making the contact. Time required for the beam to make the contact can be derived exploiting simple Newtonian mechanics. The beam motion with actuation signal at a specific force is illustrated below (Figure 5).

Suppose the beam is accelerated at a m/s^2^ at the applied force *F_a_*. Then, from the Newton’s second law of motion, the beam’s acceleration can be deduced as,

(14) $$a=\;\frac{F_{a}}{m}\;\;$$

where $F_{a}$ is the applied force and m is the mass of Si. Now, suppose the beam is displaced by *x* meter at that applied force from its initial position. Using the Newton’s equation of motions, beam displacement can be expressed as,

(15) $$x=\;v_{0}t+\;\frac{1}{2}at^{2}\;\;$$

Here, $v_{0}$ is the beam’s initial velocity, t is the time to make contact, and x is the distance travelled by the beam. The beam is initially stable, so $v_{0}$ = 0. Now, rearranging the equation for t and replacing a using Equation (14), we have,

(16) $$t_{b}=\sqrt{\frac{2mx}{F_{a}}}\;$$

As such, the total time needed to make the contact can be calculated as,

(17) $$T=t_{r}+t_{b}$$

**Figure 6:**
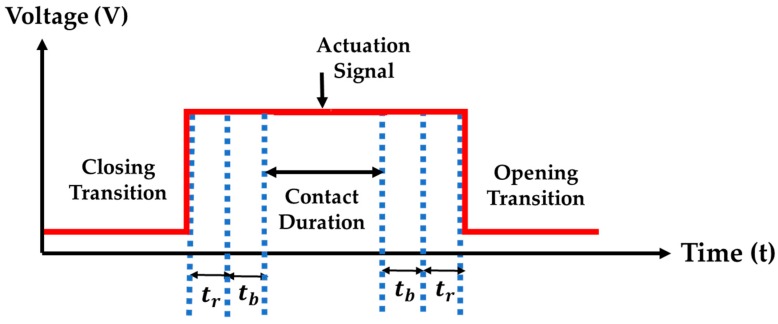
Typical timing diagram associated with microcontact actuation signal. The red pulse represents the time elapsed by an actuation signal, the first dotted blue line represents the time delay associated with the piezoelectric actuator ($t_{r}$), and the second dotted blue line represents mechanical contact closure time associated with a Si beam ($t_{b}$).

Now, if we consider a square wave with 50% duty cycle as an actuation signal, then the timing diagram can be illustrated as Figure 6.

Now, the maximum frequency of an actuation signal can be determined as,

(18) $$f=\;\frac{1}{2T}$$

**Figure 7:**
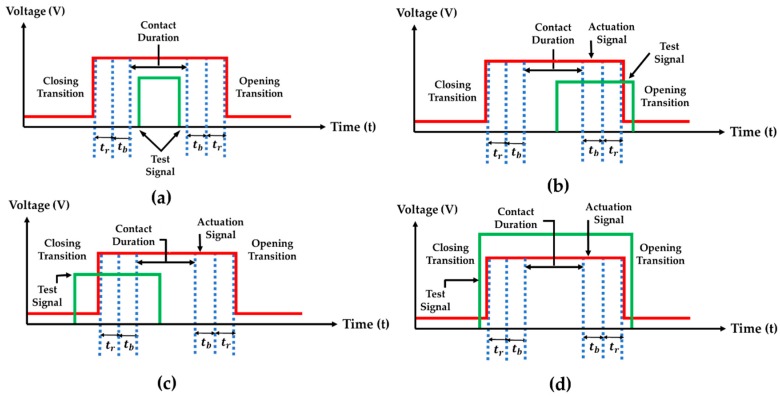
Illustrations of relative timing with respect to test signal: (**a**) Cold-switching, (**b**) hot closure, (**c**) hot opening, and (**d**) hot-switching.

In the test fixture, we could control the actuator’s cycling rate as well as the frequency of the test signal. The contact was subjected to either cold-switching or hot-switching depending on the phase and contact duration (Figure 7).

Cold switching implies that the test signal flowed only through the contact during the fully contact closure period, as shown in Figure 7a. Hot-switching implies the situation where the test signal is applied into the contact from before the contact closure to after the opening transition, as shown in Figure 7b–d. The study of contact surface tribology under these cycling situations is very critical for the enhancement of microcontact reliability for future robust microswitch design.

In order to characterize the development of contact surfaces, we performed an initial contact test (ICT), a cold-switching test (CST), and a hot-switching test (HST). For the ICT test, a known DC load was applied to the beam, and the integrated force sensor was advanced slowly in 20 nm increments until the sensor tip contacted the beam. We continued to advance the force sensor until the current through and voltage drop across the contact was observed. At this time, the force sensor was zeroed, and the actuator was advanced ~300 nm to obtain the desired contact forces (e.g., ~20–100 µN), depending on the specific contact support structure being used. The controlling of actuator step size, time interval, and applied force, as well as the data recording for applied currents and measured voltages, were performed through intensive LabView programming.

During CST testing, a signal was applied and/or removed to the contact only when the contact remained fully closed. At this stage, the measurement of contact current and voltage was made to determine the contact resistance. After finishing the measurements, the applied current was turned off to open the contact. This test was performed for the desired number of contact cycles.

During HST, the test signal was applied to the contact when the contact was fully opened or transitioned open/close. LabView software was used to control the testing for acquiring the contact resistance, force, and other associated measurements.

## 3. Proposed Improved Microcontact Support Structure Fabrication Process

**Figure 8:**
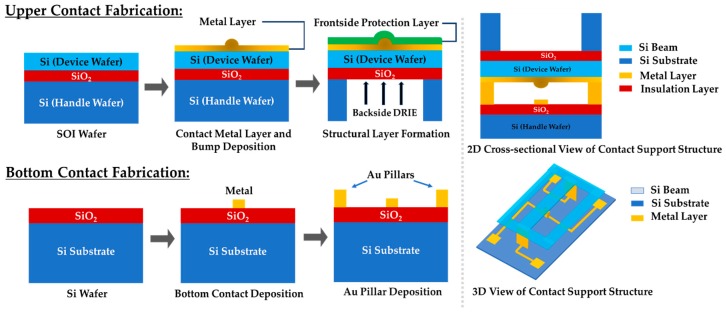
Proposed microcontact support structure fabrication process.

In this work, we fabricated two separate microcontacts through an SOI micromachined process to mimic an actual MEMS switch. Figure 8 shows the schematic diagram of our proposed device structure.

The microcontact support structure was fabricated to facilitate the Holm cross-bar measurement configuration. In this structure, two contacts were made at the two ends of the fixed-fixed beam, and the other two contacts were made at the both sides of the lower contact, as shown in the top right of Figure 8. This Holm cross-bar contact support structure facilitated the simultaneous measurements of contact resistance associated with test material and the contact force applied to the beam. During alignment time, the tip of the force sensor was positioned directly on top of the beam so that the piezoelectric actuator can close the contact smoothly and perfectly. 

### 3.1. Upper Contact Fabrication 

**Figure 9:**
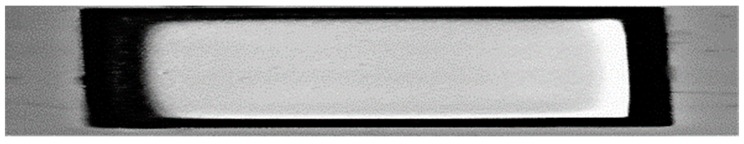
Deep reactive ion etching (DRIE) for structural layer formation.

The contact support structure was fabricated using both the surface and bulk micromachining process of a silicon on insulator (SOI) wafer. The SOI wafer consists of a 500 µm thick handle wafer (resistivity ~1000 Ω-cm), 2 µm thick buffered oxide layer, and a 5 µm thick device layer (resistivity ~1000 Ω-cm). Initially, the contact layer metallization was performed. A series of hemispherical upper contact bumps, ranging in size from 2–8 μm in diameter, were patterned and deposited. A planar circular pattern was converted into a hemispherical shape by partial heating reflow at high temperature. Afterwards, a 300 nm gold (Au) planar metal contact layer was deposited through the sputtering process. Right before that, a 10 nm titanium (Ti) adhesion layer was deposited. Then, the device layer was patterned and etched down to buried oxide layer through reactive ion etching (RIE) process to attain the bridge structure. This Si layer with sputtered gold (Au) metal layer was exploited as a structural layer and was temporarily covered by a protection layer during the backside deep reactive ion etching (DRIE) Bosch process (Figure 9). A cavity was formed within the handle wafer by implementing DRIE to facilitate to release the switching structure. Moreover, it provided space for the force sensor for contact force measurement. Lastly, the buried oxide (BOX) layer of the SOI wafer was etched out from the handle side of the wafer by RIE. In this way, the upper contact support structure was released.

### 3.2. Bottom Contact and Contact Pad Fabrication 

All the bottom contacts, signal traces, and contact pads were fabricated on a silicon substrate isolated by either silicon oxide or silicon nitride. A series of planar contacts, ranging in size from 6–12 µm were fabricated using a standard lift-off process with 300 nm thick evaporated Au, along with a 20 nm of titanium (Ti) adhesion layer. 

### 3.3. Bottom Metal Pillar Fabrication

**Figure 10:**
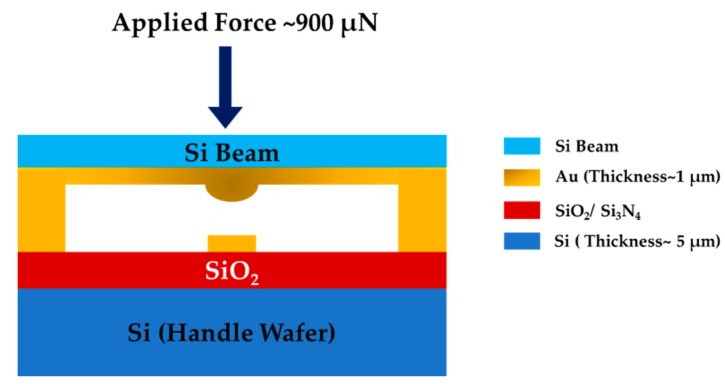
Schematic model for determining Au pillar dimensions.

To enable switching between upper hemisphere and lower planar contacts, we fabricated gold (Au) contact pillars about 1 µm high on top of the bottom Si wafer. As the beam was loaded at the center, the force was symmetrically distributed (~450 µN) at the both pillars (Figure 10). 

Now, the stress on the pillar can be calculated as,

(19) $$\sigma =\;\frac{F}{A}=\;\frac{450\;µN}{A}$$

where *F* is the applied force and *A* is the cross-sectional area of the pillar. Yield strength for Au, $\sigma_{Y}=20–205\;\mathrm{MPa}$ [25]. To make a stable pillar at that applied force, the stress should not be greater than the minimum yield point. Thus,

$$\mathrm{Stress}\;\mathrm{on}\;\mathrm{Au}\;\mathrm{pillar}\;<\sigma_{Y}\;\frac{450\;µN}{A}<\;20\;\mathrm{MPa}\;A\;>\;22.5\times 10^{-12}\;m^{2}$$

Now, if we fix the width as 75 µm, then the condition on length can be expressed as:

$$L\;>\;300\times 10^{-9}\;m\;L\;>\;300\;\mathrm{nm}$$

As such, a gold pillar of length more than 300 nm was strong enough to hold the beam at available applied force in the test setup.

**Figure 11:**
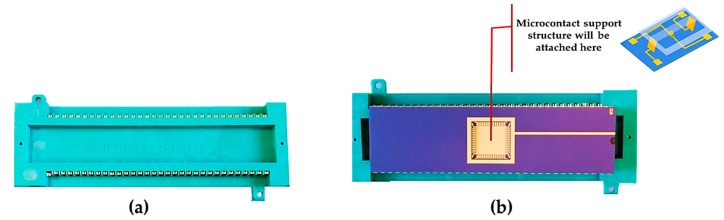
(**a**) Carrier socket; (**b**) package placed into the carrier socket.

After fabricating the microcontact support structure, it was attached to a package using crystal bonder. Then, we wire bonded it to the package. In this way, we eradicated the probe requirement. Besides, it helped us to precisely measure the voltage and current across the contacts. Next, we put the package into a carrier socket. The socket had pins for each of the wire bonds. Figure 11 shows a socket and a socket with package.

## 4. Results and Discussion

### 4.1. Impact of Beam Geometry on Stiffness, Resonance Frequency, and Contact Closure Time

As contact sliding occurs during switching the cantilever type beam structure [3], we chose the fixed-fixed beam structure for our test fixture. The beam geometry has been varied to obtain the appropriate stiffness for the available force. The main purpose of our test fixture was to investigate the microcontact reliability and performance. To evaluate contact properties (contact resistance, contact voltage, and contact temperature), we have used well established analytical models associated with the microcontact. As the beam’s properties have lesser impact on microcontact reliability, we estimated its mechanical properties using Euler-Bernoulli beam theory and SOLIDWORKS [26].

**Figure 12:**
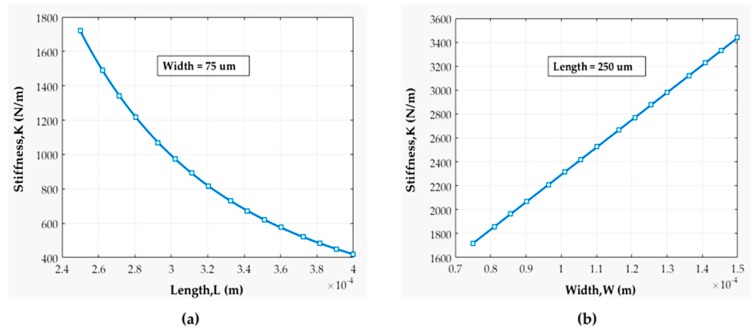
Stiffness variation with respect to (**a**) beam’s length and (**b**) beam’s width.

**Figure 13:**
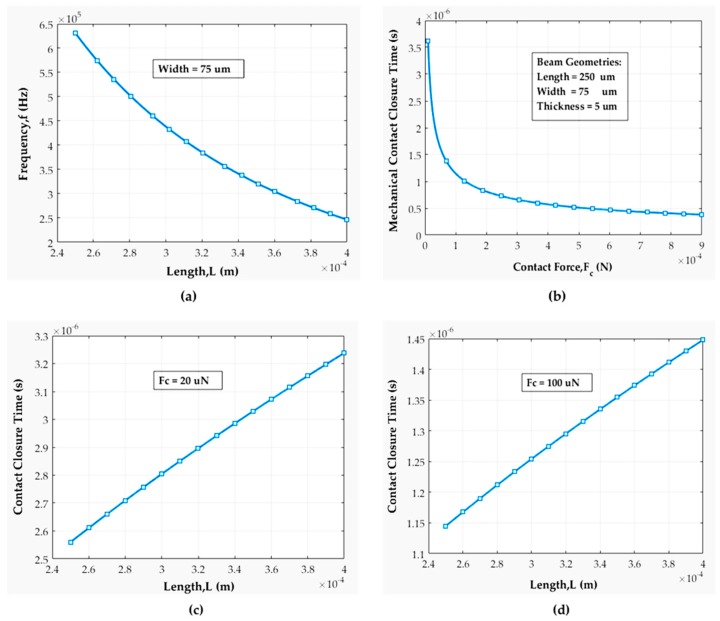
(**a**) Resonance frequency with beam’s geometry; (**b**) mechanical contact closure time with available contact force; (**c**) mechanical contact closure time versus beam length for 20 µN contact force; (**d**) mechanical contact closure time versus beam length for 100 µN contact force.

Figure 12 depicts that the stiffness of a fixed-fixed beam is highly dependent on its length rather than width. We fabricated beam lengths varied from 250–400 µm with stiffness ranging from 400–1800 N/m. The results indicate that during beam fabrication we should keep the beam width fixed but vary the beam length. Figure 13a shows the variation of the beam’s resonance frequency with its lengths for a fixed width. The resonance frequency is distinct for every beam length. Frequency decreased from 250 kHz to 625 kHz with the increase of beam length. Therefore, the actuation frequency should not have exceeded 625 kHz for our beam structure. In Figure 13b, the relation between mechanical contact closure time and contact force is revealed. Generally, the contact force for a metal to metal series MEMS switch is around 50–200 μN to ensure very low contact resistance [1,2,3]. Hence, all the beam and contact mechanics analyzed here are over 10–1000 µN contact force. 

The high contact force results in very short contact closing time and it is calculated as 1 μs at 100 μN.

Figure 13c,d show the impact of beam length on mechanical contact closure time. For 20 µN contact force, the highest contact closure time was found to be 3.25 µs. For 100 µN contact force, the highest contact closure time was 1.45 µs for the longest beam. The results imply that the mechanical contact closure time doesn’t vary much more with geometry than with the contact force. In addition, we have analyzed the piezoelectric actuator’s (Thorlabs’ PAZ005) response time under various circuit conditions.

**Figure 14:**
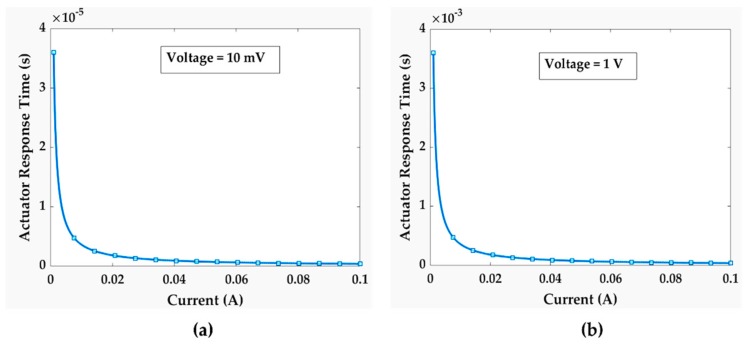
Actuator response time under different circuit conditions; (**a**) 10 mV; (**b**) 1 V.

From the plots in Figure 14, we get to know that maximum actuator response time is in the range of milliseconds. However, we previously found that mechanical contact closure time is in the range of only few microseconds. As the piezoelectric actuator’s response time is much longer than the mechanical contact closure time, the total contact closure time, and hence the contact cycling rate, will be dominated by the actuator’s response time. We investigated cold switching and hot-switching effects under various circuit conditions based on the relative timing between the contact closure time and test signal.

We designed our fixed-fixed beam structure and performed finite element analysis (FEA) simulations to investigate the mechanical responses of the Si beam using the SOLIDWORKS mechanical simulation module [26]. The parameters involved in the simulation were beam geometries (i.e., beam length, L; beam width, W; beam thickness, t) and physical properties of Si. The simulations were done for a wafer of Si (100) plane. The following subsections (Section 4.1.1, Section 4.1.2, and Section 4.1.3) contain stress, displacement, and contact force analysis of doubly fixed beams having different geometries.

#### 4.1.1. Stress Analysis

**Figure 15:**
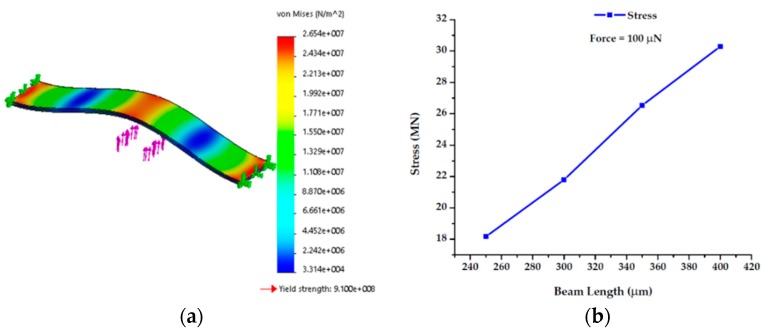
(**a**) Stress analysis of beam length under point force; (**b**) beam length versus Stress at 100 µN force.

General stress state description was given in a scale of von Mises stress number. It gives an overall idea about the stress state at a particular location in the beam. Figure 15 illustrates the von Mises stress distribution for a doubly fixed beam. Results show that a beam with length 350 µm and width 75 µm had highest stress at the center and at the edges (Figure 15a). It happened because a load about 100 µN was placed at the center of the beam, and the two edges were fixed at the end. Interestingly, it is also evident from the simulation that the load was symmetrically distributed at the edges and that stress on the beam increases as we increased the beam length (Figure 15b). For all these cases, yield strength was much higher than the stress generated due to applied force. Therefore, the device can properly operate within this applied force range.

#### 4.1.2. Displacement Analysis

**Figure 16:**
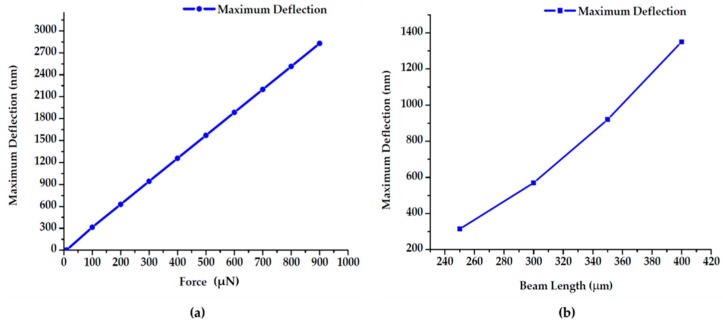
(**a**) Force versus maximum deflection; (**b**) beam length versus maximum deflection.

Figure 16 represents displacements of a doubly fixed beams of 250 µm long and 75 µm wide at 10–900 µN force. At this range of force, maximum displacement was seen at beam’s center region and it can expand from ~250–2800 nm (Figure 16a).

Furthermore, we performed FEA for displacement with respect to beam length at 100 µN force (Figure 16b). The result suggests that we can tune the maximum displacement by altering either beam length or applied force. However, in our experimental setup, the contact gap was around 300 nm. Therefore, we did simulations to find out appropriate force to achieve our desired 300 nm deflection. 

#### 4.1.3. Force Tuning for Different Length’s Beam

**Figure 17:**
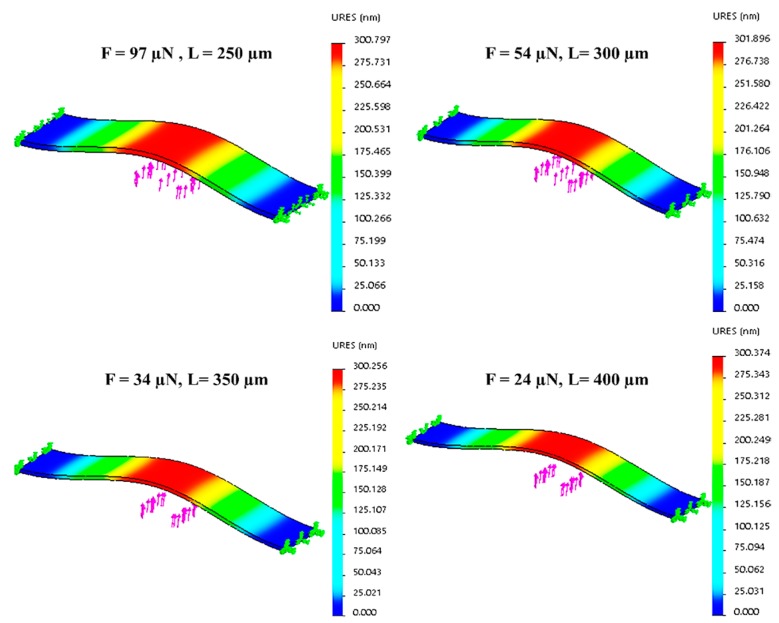
Force tuning and displacement analysis for different beam length under point force.

We attained ~300 nm displacement by considering different beam length and applied force values. The fixed-fixed beam’s maximum displacement due to single point load at center can be expressed as [27,28,29],

(20) $$\delta_{max}=\frac{FL^{3}}{192EI}\;\;$$

where $\delta_{max}$ stands for displacement, *F* represents point load, *L* is the beam’s length, *E* is the modulus of elasticity, and *I* refers to the area moment of inertia. Equation (20) indicates that the displacement is a function of both applied force and beam length. Hence, we can get a specific maximum displacement value for different sets of applied force and beam length values (Figure 17).

**Figure 18:**
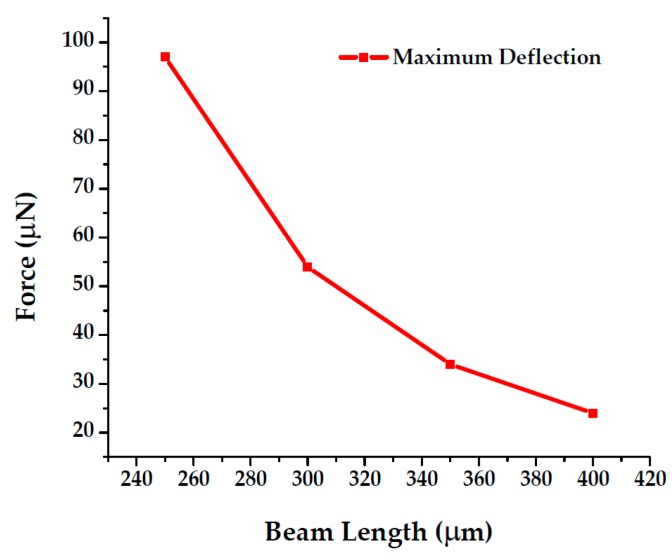
Relation between force and beam length for constant displacement.

We found an applied force from 24–97 µN is required to get ~300 nm displacement for our available beam lengths (Figure 18). 

#### 4.1.4. Contact Resistance, Voltage and Temperature Analysis with Contact Force

**Figure 19:**
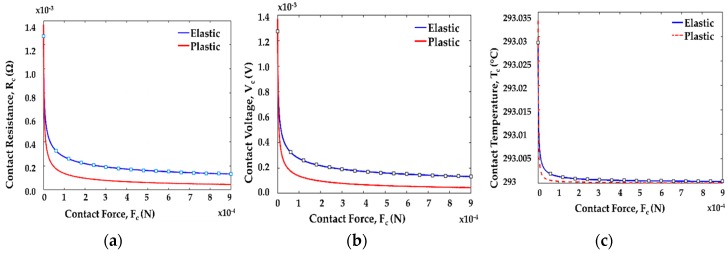
(**a**) Contact resistance; (**b**) contact voltage; (**c**) contact temperature of a single gold (Au) asperity contact for elastic and plastic region.

Figure 19 shows that the contact resistance, contact voltage, and contact temperature decrease and become stable at the higher force region. Contact temperature decreased negligibly with increasing contact force. Initially, gold on gold (Au/Au) microcontact was tested and compared with these theoretical results to validate our contact support model. Afterwards, a wide range of contact materials was investigated with this contact structure. It showed that the contact resistance, contact voltage, and contact temperature decreased and became stable at the higher force region. 

**Figure 20:**
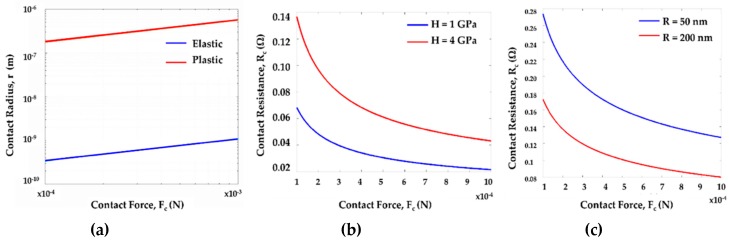
(**a**) Contact radius of a single gold (Au) asperity for elastic and plastic region; (**b**) contact resistance with varied hardness for a gold (Au) “a-spots”; (**c**) contact resistance of a single gold (Au) asperity contact with varied radius of curvature.

Figure 20a depicts the relation between contact radius and contact force associated with a single gold (Au) asperity contact spot for the elastic and plastic region. We have studied the variation of contact radius from the low contact force region (~100 µN) to the high contact force region (~1000 µN), as most of the MEMS switches find their applications in this range. It is evident from Figure 20 that a single asperity contact spot with radius of approximately 100 nm requires at least 100’s of µN force to make a stable contact. 

The effect of hardness and radius of curvature of contact materials on contact resistance is depicted in Figure 20b,c. Results show the variation of contact resistance with the variation of microcontact’s radius of curvature. Radius of curvature’s variation can come from the variations in deposition process or release process or from the quality of the metal film during contact fabrication process. The decrease in radius of curvature results in an increased contact resistance within the region of interest for contact force. This change occurs in the contact material’s elastic region. To obtain a stable contact resistance, an increased radius of curvature requires an increased amount of contact force.

Table 1 summarizes the performance parameters of similar microcontact test fixtures (i.e., MEMS/AFM/SPM) and our proposed test fixture. It shows that unlike other test fixture approaches, the test fixture presented here can provide both high cycle rates and the simultaneous measurement of contact force and resistance with nN force resolution.

## 5. Conclusions

In this work, we designed a unique microcontact support structure for improved post-mortem analysis. Simulation results for contact resistance, contact voltage, and contact temperature have been demonstrated based on theoretical models. Mechanical responses of fixed-fixed beam structure were explored via finite element analysis simulations. Moreover, a relation between the contact closure time and contact force was established for the fixed-fixed beam structure. Closure time’s impact on cold-switching and hot-switching was studied with respect to the test signal. Furthermore, a condition for the minimum length of an Au pillar was derived for the available applied force. All these simulated results will be compared with our newly fabricated gold on gold (Au/Au) contact support structure. Subsequently, a wide variety of other potential contact materials will be inspected to find their appropriate contact force, contact resistance, adhesion, and other unexplored physics relevant to MEMS switching applications. The microcontact support structure will be fabricated through SOI micromachining technique. This unique method will allow a very simple, fast, and efficient postmortem analysis related to the microcontact surface tribology. In addition, engineered micro-electrical contacts will be examined using our novel test fixture for obtaining significant data to design a future robust and reliable MEMS switch.

## Figures and Tables

**Table 1:** Performance comparison between proposed and conventional test fixtures.

Test Fixtures	Cycle Rate	Contact Force Measurement	Force Resolution
Proposed Test Fixture	~5 KHz [30]	Directly measured	nN [30]
MEMS/ AFM/ SPM	~10–100 Hz [9,10,11,12]	Inferred from model	μN [2]
